# Noise Reduction for CFA Image Sensors Exploiting HVS Behaviour

**DOI:** 10.3390/s90301692

**Published:** 2009-03-10

**Authors:** Angelo Bosco, Sebastiano Battiato, Arcangelo Bruna, Rosetta Rizzo

**Affiliations:** 1 STMicroelectronics, Stradale Primosole 50, 95121 Catania, Italy; E-Mail: arcangelo.bruna@st.com; 2 Università di Catania, Dipartimento di Matematica ed Informatica, Viale A. Doria 6, 95125 Catania, Italy; E-Mails: battiato@dmi.unict.it; rosetta.rizzo@dmi.unict.it

**Keywords:** Noise Reduction, Color Filter Array, HVS, Texture Detection

## Abstract

This paper presents a spatial noise reduction technique designed to work on *CFA* (Color Filtering Array) data acquired by *CCD/CMOS* image sensors. The overall processing preserves image details using some heuristics related to the *HVS* (Human Visual System); estimates of local texture degree and noise levels are computed to regulate the filter smoothing capability. Experimental results confirm the effectiveness of the proposed technique. The method is also suitable for implementation in low power mobile devices with imaging capabilities such as camera phones and *PDAs*.

## 1. Introduction

The image formation process through consumer imaging devices is intrinsically noisy. This is especially true using low-cost devices such as mobile-phones, PDAs, etc., mainly in low-light conditions and the absence of flash-guns [1].

The final perceived quality of images acquired by digital sensors can be optimized through multi-shot acquisitions (e.g., extending dynamic range [2], increasing resolution [3]) and/or using *ad-hoc* post-processing techniques [4,5] taking into account the raw data acquired by Bayer matrixed image sensors [6]. These are grayscale sensors covered by *CFA* (Color Filter Array) to enable color sensitivity, such that each cell of the sensor array is receptive to only one color component. The final color image is obtained by means of a color reconstruction (*demosaicing*) algorithm that combines the color information of neighboring pixels [7–9] and [10]. A useful review of technology and methods in the field can be found in [1] and [11].

In this paper we propose a novel spatial noise reduction method that directly processes the raw *CFA* data, combining together *HVS* (Human Visual System) heuristics, texture/edges preservation techniques and sensor noise statistics, in order to obtain an effective adaptive denoising.

The proposed algorithm introduces the concept of the usage of *HVS* peculiarities directly on the *CFA* raw data from the sensor. In addition, the complexity of the algorithm is kept low by using only spatial information and a small fixed-size filter processing window, allowing real-time performance on low cost imaging devices (e.g., mobile phones, PDAs).

The *HVS* properties, able to characterize or isolate unpleasant artifacts, are complex (highly nonlinear) phenomena not yet completely understood involving a lot of complex parameters [12,13]. Several studies in the literature have tried to simulate and code some known aspects in order to find reliable image metrics [14–16] and heuristics to also be applied for demosaicing [17].

Sophisticated denoising methods such as [18–20] perform multiresolution analysis and processing in the wavelet domain. Other techniques, as suggested in [21], use anisotropic non-linear diffusion equations, but work iteratively. Spatial denoising approaches having texture discrimination capabilities can be found in [1,23,24], whereas methods implementing texture discrimination using fuzzy logic are described in [25,26]. Other kinds of noise, such as *fixed pattern noise* (*FPN*) can be treated *ad-hoc*, in [27] a method suitable is presented.

The proposed filtering method is a trade-off between real time implementation with very low hardware logic and the usage of some *HVS* peculiarities, texture and noise level estimation. The filter adapts its smoothing capability to local image characteristics yielding effective results in terms of visual quality.

The paper is structured as follows: in the next section some details about the *CFA* and *HVS* characteristics are briefly discussed; in Section 3 the overall details of the proposed method are presented. An experimental section reports the results and some comparisons with other related techniques. The final section tracks directions for future works.

## 2. Background

### 2.1. Bayer Data

In typical imaging devices a color filter is placed on top of the imager making each pixel sensitive to only one color component. A color reconstruction algorithm interpolates the missing information at each location and reconstructs the full *RGB* image [9–11]. The color filter selects the red, green or blue component for each pixel; this arrangement is known as Bayer pattern [6]; other arrangements of *CFA* data take into account *CMY* complementary colors, but the *RGB* color space is the most common.

**Figure 1.**
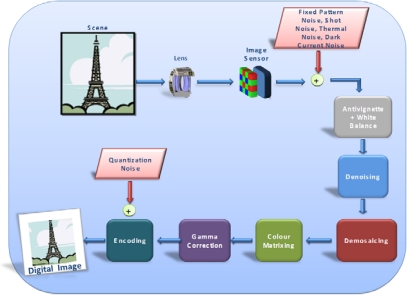
Image Generation Pipeline.

The number of green elements is twice the number of red and blue pixels due to the higher sensitivity of the human eye to the green light, which, in fact, has a higher weight when computing the luminance. The proposed filter processes raw Bayer data, providing the best performance if executed as the first algorithm of the IGP (Image Generation Pipeline). A typical image reconstruction pipeline is shown in Figure 1.

### 2.2. Basic Concepts about the Human Visual System

It is well known that the *HVS* has a different sensitivity at different spatial frequencies [28]. In areas containing mean frequencies the eye has a higher sensitivity. Furthermore, chrominance sensitivity is weaker than the luminance one.

*HVS* response does not entirely depend on the luminance value itself, rather, it depends on the luminance local variations with respect to the background; this effect is described by the Weber-Fechner’s law [13,29], which determines the minimum difference *DY* needed to distinguish between *Y* (background) and *Y+DY*. Different values of *Y* yield to different values of *DY*.

The aforementioned properties of the *HVS* have been used as a starting point to devise a *CFA* filtering algorithm. Luminance from *CFA* data can be extracted as explained in [30], but for our purposes it can be roughly approximated by the green channel values before gamma correction.

The filter changes its smoothing capability depending on the *CFA* color of the current pixel and its similarity with the neighborhood pixels.

More specifically, in relation to image content, the following assumptions are considered:

- if the local area is homogeneous, then it can be heavily filtered because pixel variations are basically caused by random noise.
- if the local area is textured, then it must be lightly filtered because pixel variations are mainly caused by texture and by noise to a lesser extent; hence only the little differences can be safely filtered, as they are masked by the local texture.

## 3. The Proposed Technique

### 3.1. Overall filter block diagram

**Figure 2.**
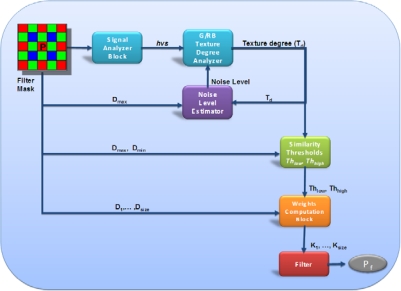
Overall Filter Block Diagram.

A block diagram describing the overall filtering process is illustrated in Figure 2. Each block will be separately described in detail in the following sections.

The fundamental blocks of the algorithm are:

- ***Signal Analyzer Block***: computes a filter parameter incorporating the effects of human visual system response and signal intensity in the filter mask.
- ***Texture Degree Analyzer***: determines the amount of texture in the filter mask using information from the *Signal Analyzer Block.*
- ***Noise Level Estimator***: estimates the noise level in the filter mask taking into account the texture degree.
- ***Similarity Thresholds Block***: computes the fuzzy thresholds that are used to determine the weighting coefficients for the neighborhood of the central pixel.
- ***Weights Computation Block***: uses the coefficients computed by the *Similarity Thresholds Block* and assigns a weight to each neighborhood pixel, representing the degree of similarity between pixel pairs.
- ***Filter Block***: actually computes the filter output.

The data in the filter mask passes through the *Signal Analyzer* block that influences the filter strength in dark and bright regions (Section 3.2 for further details). The *HVS* value is used in combination with the output of the *Texture Degree Analyzer* (Section 3.4) and *Noise Level Estimator* (Section 3.5) to produce the similarity thresholds used to finally compute the weights assigned to the neighborhood of the central pixel (Section 3.6). The final filtered value is obtained by a weighted averaging process (Section 3.7).

### 3.2. Signal Analyzer Block

As noted [31–33], it is possible to approximate the minimum intensity gap that is necessary for the eye to perceive a change in pixel values. The base sensitivity thresholds measure the contrast sensitivity in function of frequency while fixing the background intensity level. In general, the detection threshold varies also with the background intensity. This phenomenon is known as luminance masking or light adaptation. Higher gap in intensity is needed to perceive a visual difference in very dark areas, whereas for mid and high pixel intensities a small difference in value between adjacent pixels is more easily perceived by the eye [32].

It also crucial to observe that in data from real image sensors, the constant *AWGN (Additive White Gaussian Noise)* model does not fit well the noise distribution for all pixel values. In particular, as discussed in [34], the noise level in raw data is predominantly signal-dependent and increases as the signal intensity raises; hence, the noise level is higher in very bright areas. In [34] and [35] it is also illustrated how clipping in data is the cause of noise level underestimation; e.g., noise level for pixels close to saturation cannot be robustly tracked because the signal reaches the upper limit of the allowed bitdepth encoding.

**Figure 3.**
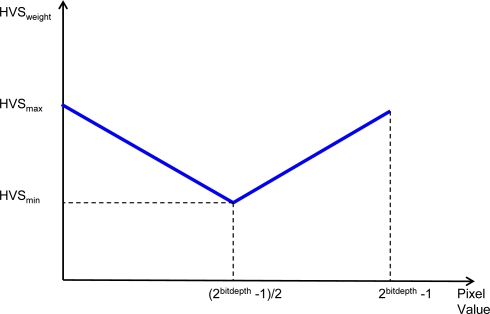
HVS curve used in the proposed approach.

We decided to incorporate the above considerations of luminance masking and sensor noise statistics into a single curve as shown in Figure 3. The shape of this curve allows compensating for lower eye sensitivity and increased noise power in the proper areas of the image, allowing adaptive filter smoothing capability in relation to the pixel values.

A high HVS value (HVSmax) is set for both low and high pixel values: in dark areas the human eye is less sensitive to variations of pixel intensities, whereas in bright areas noise standard deviation is higher. HVS value is set low (HVSmin) at mid pixel intensities.

As stated in Section 2.2, in order to make some simplifying assumptions, we use the same HVS curve for all CFA colour channels taking as input the pixel intensities directly from the sensor. The HVS coefficient computed by this block is used by the Texture Degree Analyzer that outputs a degree of texture taking also into account the above considerations (Section 3.4).

### 3.3. Filter Masks

**Figure 4.**
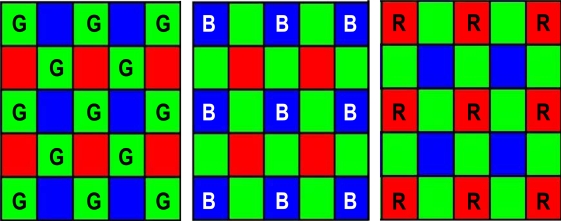
Filter Masks for Bayer Pattern Data.

The proposed filter uses different filter masks for green and red/blue pixels to match the particular arrangement of pixels in the *CFA* array. The size of the filter mask depends on the resolution of the imager: at higher resolution a small processing window might be unable to capture significant details. For our processing purposes a 5×5 window size provided a good trade-off between hardware cost and image quality, allowing us to process images up to 5 megapixels, a resolution that is typical of high end mobile phones. Typical Bayer processing windows are illustrated in Figure 4.

### 3.4. Texture Degree Analyzer

**Figure 5.**
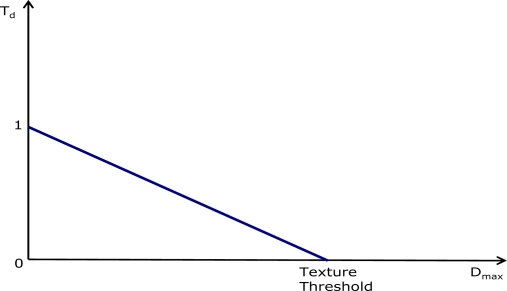
Green Texture Analyzer.

The texture analyzer block computes a reference value *T_d_* that is representative of the local texture degree. This reference value approaches 1 as the local area becomes increasingly flat and decreases as the texture degree increases (Figure 5). The computed coefficient is used to regulate the filter smoothing capability so that high values of *T_d_* correspond to flat image areas in which the filter strength can be increased.

**Figure 6.**
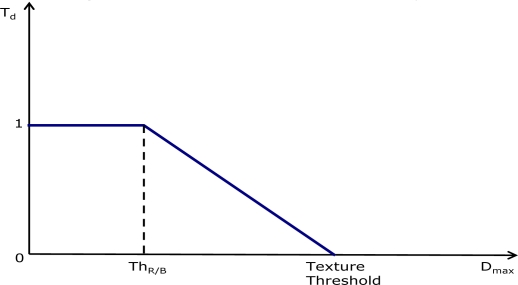
Red/Blue texture analyzer.

Depending on the color of the pixel under processing, either green or red/blue, two different texture analyzers are used. The red/blue filter power is increased by slightly modifying the texture analyzer making it less sensitive to small pixel differences (Figure 6). The texture analyzer block output depends on a combination of the maximum difference between the central pixel and the neighborhood *D_max_* and *TextureThreshold*, a value that is obtained by combining information from the *HVS* response and noise level, as described below (2).

The green and red/blue texture analyzers are defined as follows:

(1) $$\begin{array}{l} T_{d}\;\left(\mathrm{green}\right)=\left\{ \begin{array}{ll} 1 & D_{\text{max}}=0\\ -\frac{D_{\text{max}}}{\mathrm{TextureThreshold}}+1 & 0<D_{\text{max}}\leq \mathrm{TextureThreshold}\\ 0 & D_{\text{max}}>\mathrm{TextureThreshold} \end{array}\right.\\ T_{d}\;\left(\mathrm{red}/\mathrm{blue}\right)=\left\{ \begin{array}{ll} 1 & D_{\text{max}}\leq {\mathrm{Th}}_{R/B}\\ -\frac{\left(D_{\text{max}}-{\mathrm{Th}}_{R/B}\right)}{\left(\mathrm{TextureThreshold}-{\mathrm{Th}}_{R/B}\right)}+1 & {\mathrm{Th}}_{R/B}<D_{\text{max}}\leq \mathrm{TextureThreshold}\\ 0 & D_{\text{max}}>\mathrm{TextureThreshold} \end{array}\right. \end{array}$$

hence:

- if *T_d_* = 1 the area is assumed to be completely flat;
- if 0 < *T_d_* < 1 the area contains a variable amount of texture;
- if *T_d_* = 0, the area is considered to be highly textured.

The texture threshold for the current pixel, belonging to Bayer channel *c* (*c=R,G,B*)*,* is computed by adding the noise level estimation to the *HVS* response (2):

(2) $${\mathrm{TextureThreshold}}_{c}\;(k)={\mathrm{HVS}}_{\mathrm{weight}}(k)+{\mathrm{NL}}_{c}(k-1)$$

where *NL_c_* denotes the noise level estimation on the previous pixel of the same Bayer color channel *c*(see Section 3.5) and *HVS_weight_* (Figure 3) can be interpreted as a *jnd* (*just noticeable difference*); hence an area is no longer flat if the *D_max_* value exceeds the *jnd* plus the local noise level *NL*.

**Figure 7.**
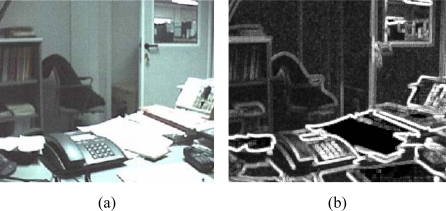
Texture Analyzer output: (a) input image after colour interpolation (b) gray-scale texture degree output: bright areas correspond to high frequency, dark areas correspond to low frequencies.

The green texture analyzer (Figure 5) uses a stronger rule for detecting flat areas, whereas the red/blue texture analyzer (Figure 6) detects more flat areas, being less sensitive to small pixel differences below the *Th_R/B_* threshold. The gray-scale output of the texture detection is shown in Figure 7: bright pixels are associated to high texture, dark pixels to flat areas.

### 3.5. Noise Level Estimator

In order to adapt the filter smoothing capability to the local characteristics of the image, a noise level estimation is required. The proposed noise estimation solution is pixel based and is implemented taking into account the previous estimation to calculate the current one.

The noise estimation equation is designed so that:

- if the local area is completely flat (*T_d_* = 1), then the noise level is set to *D_max_*;
- if the local area is highly textured (*T_d_* = 0), the noise estimation is kept equal to the previous region (i.e., pixel);
- otherwise a new value is estimated.

Each color channel has its own noise characteristics hence noise levels are tracked separately for each color channel. The noise level for each channel is estimated according to the following formulas:

(3) $$\begin{array}{l} {\mathrm{NL}}_{R}\;(k)=T_{d}\;(k)*D_{\text{max}}\;(k)+[1-T_{d}\;(k)]*{\mathrm{NL}}_{R}\;(k-1)\\ {\mathrm{NL}}_{G}\;(k)=T_{d}\;(k)*D_{\text{max}}\;(k)+[1-T_{d}\;(k)]*{\mathrm{NL}}_{G}\;(k-1)\\ {\mathrm{NL}}_{B}\;(k)=T_{d}\;(k)*D_{\text{max}}\;(k)+[1-T_{d}\;(k)]*{\mathrm{NL}}_{B}\;(k-1) \end{array}$$

where *T_d_(k)* represents the texture degree at the current pixel and *NL_c_(k*−*1)* (*c=R,G,B*) is the previous noise level estimation, evaluated considering pixel of the same colour, already processed. For *k* = 1 the values *NL_R_(k*−*1)*, *NL_G_(k*−*1)* and *NL_B_(k*−*1)* are set to an initial low value depending on the pixel bit-depth. These equations satisfy requirements i), ii) and iii). The raster scanning order of the input image is constrained by global HW architecture. Starting from different spatial locations the noise level converges to the same values due to the presence of homogeneous areas that are, of course, prominent in almost all natural images.

### 3.6. Similarity Thresholds and Weighting Coefficients computation

**Figure 8.**
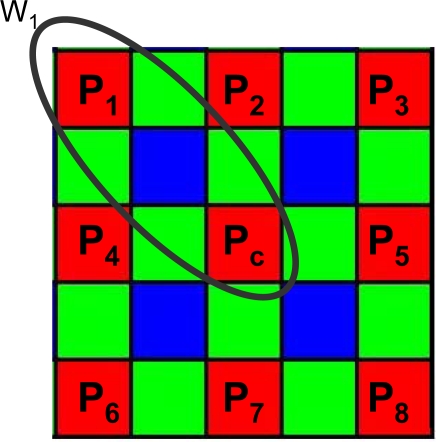
The Wi coefficients weight the similarity degree between the central pixel and its neighborhood.

The final step of the filtering process consists in determining the weighting coefficients *W_i_* to be assigned to the neighboring pixels of the filter mask. The absolute differences *D_i_* between the central pixel and its neighborhood must be analyzed in combination with the local information (noise level, texture degree and pixel intensities) for estimating the degree of similarity between pixel pairs (see Figure 8). As stated in Section 2.2, if the central pixel *P_c_* belongs to a textured area, then only small pixel differences must be filtered. The lower degree of filtering in textured areas allows maintaining the local sharpness, removing only pixel differences that are not perceived by the *HVS*.

**Figure 9.**
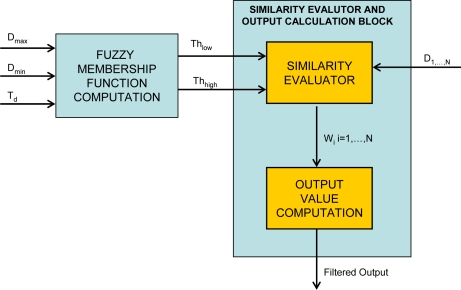
Block diagram of the fuzzy computation process for determining the similarity weights between the central pixel and its N neighborhoods.

The process for determining the similarity thresholds and the *W_i_* coefficients can be expressed in terms of fuzzy logic (Figure 9).

Let:

- *P_c_* be the central pixel of the working window;
- *P_i_*, i = 1,…,7, be the neighborhood pixels;
- *D_i_* = *abs(P_c_* − *P_i_),* i=1,…,7 the set of absolute differences between the central pixel and its neighborhood;

In order to obtain the *W_i_* coefficients, each absolute difference *D_i_* must be compared against two thresholds *Th_low_* and *Th_high_* that determine if, in relation to the local information, the *i-th* difference *D_i_* is:

- small enough to be heavily filtered,
- big enough to remain untouched,
- an intermediate value to be properly filtered.

The two thresholds can be interpreted as fuzzy parameters shaping the concept of similarity between pixel pairs. In particular, the associated fuzzy member function computes the similarity degree between the central and a neighborhood pixel.

**Figure 10.**
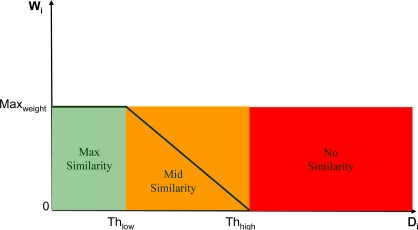
Weights assignment (Similarity Evaluator Block). The i-th weight denotes the degree of similarity between the central pixel in the filter mask and the i-th pixel in the neighborhood.

By properly computing *Th_low_* and *Th_high_*, the shape of the membership function is determined (Figure 10).

To determine which of the above cases is valid for the current local area, the local texture degree is the key parameter to analyze. It is important to remember at this point that, by construction, the texture degree coefficient (*T_d_*) incorporates the concepts of dark/bright and noise level; hence, its value is crucial to determine the similarity thresholds to be used for determining the *W_i_* coefficients. In particular, the similarity thresholds are determined to obtain maximum smoothing in flat areas, minimum smoothing in highly textured areas, and intermediate filtering in areas containing medium texture; this can be obtained by using the following rules (4):

(4) $$\left\{ \begin{array}{l} {\mathrm{Th}}_{\mathrm{low}}={\mathrm{Th}}_{\mathrm{high}}=D_{\text{max}}\;\mathrm{if}\;T_{d}=1\\ {\mathrm{Th}}_{\mathrm{low}}=D_{\text{min}}\;\mathrm{if}\;T_{d}=0\\ {\mathrm{Th}}_{\mathrm{high}}=\frac{D_{\text{min}}+D_{\text{max}}}{2}\;\mathrm{if}\;T_{d}=0\\ D_{\text{min}}<{\mathrm{Th}}_{\mathrm{low}}<{\mathrm{Th}}_{\mathrm{high}}\;\mathrm{if}\;0<T_{d}<1\\ \frac{D_{\text{min}}+D_{\text{max}}}{2}<{\mathrm{Th}}_{\mathrm{high}}<D_{\text{max}}\;\mathrm{if}\;0<T_{d}<1 \end{array}\right.$$

Once the similarity thresholds have been fixed, it is possible to finally determine the filter weights by comparing the *D_i_* differences against them (Figure 10).

To summarize, the weighting coefficient selection is performed as follows. If the *i-th* absolute difference *D_i_* is lower than *Th_low_*, it is reasonable to assume that pixels *P* and *P_i_* are very similar; hence the maximum degree of similarity *Max_weight_* is assigned to *P_i_*. On the other hand, if the absolute difference between *P* and *P_i_* is greater than *Th_high_*, it is reasonable that this difference is due to texture details, hence *P_i_* is assigned a null similarity weight. In the remaining cases, i.e. when the *i-th* absolute difference falls in the interval [*Th_low_*, *Th_high_*], a linear interpolation between *Max_weight_* and 0 is performed, allowing determining the appropriate weight for *P_i_*.

### 3.7. Final Weighted Average

Let W*_1_*,…,W*_N_* (*N*: number of neighborhood pixels) be the set of weights computed for the each neighboring element of the central pixel *P_c_*. The final filtered value *P_f_* is obtained by weighted average as follows (5):

(5) $$P_{f}=\frac{1}{N}\sum_{i=1}^{N}\left[W_{i}P_{i}+\left(1-W_{i}\right)P_{c}\right]$$

In order to preserve the original bitdepth, the similarity weights are normalized in the interval [0,1], and chosen according to equation (6):

(6) $$W_{i}=\left\{ \begin{array}{l} 1\;\mathrm{if}\;D_{i}\leq {\mathrm{Th}}_{\mathrm{low}}\\ L({\mathrm{Th}}_{\mathrm{low}},{\mathrm{Th}}_{\mathrm{high}})\;\mathrm{if}\;{\mathrm{Th}}_{\mathrm{low}}<D_{i}<{\mathrm{Th}}_{\mathrm{high}}\\ 0\;\mathrm{if}\;D_{i}\geq {\mathrm{Th}}_{\mathrm{high}} \end{array}\right.$$

where *L(Th_low_, Th_high_)* performs a simple linear interpolation between *Th_low_* and *Th_high_* as depicted in Figure 10.

## 4. Experimental Results

The following sections describe the tests performed to assess the quality of the proposed algorithm. First, a test computing the noise power before and after filtering is reported. Next some comparisons between the proposed filter and other noise reduction algorithms ([25,36,37]) are described.

### 4.1. Noise Power Test

A synthetic image was used to determine the amount of noise that the algorithm is capable to remove. Let us denote:

- **I_NOISY_**: Noisy *CFA* Pattern
- **I_FILTERED_**: Filtered *CFA* Pattern
- **I_ORIGINAL_**: Original noiseless *CFA* Pattern

According to these definitions we have:

- **I_NOISY_** − **I_ORIGINAL_ = I_ADDED_NOISE_**
- **I_FILTERED_** − **I_ORIGINAL_ = I_RESIDUAL_NOISE_**

where **I_ADDED_NOISE_** is the image containing only the noise artificially added to **I_ORIGINAL_**, whereas **I_RESIDUAL_NOISE_** is the image containing the residual noise after filtering. The noise power is computed for both **I_ADDED_NOISE_** and **I_RESIDUAL_NOISE_** according to the following formula (7):

(7) $$P={20\;\text{log}}_{10}\left(\frac{1}{\mathrm{MN}}\sum_{n=0}^{N-1}\sum_{m=0}^{M-1}I{\left(m,n\right)}^{2}\right)$$

To modulate the power of the additive noise, different values of the standard deviation of a Gaussian distribution are used. Noise is assumed to be *AWGN* (*Additive White Gaussian Noise*), with zero mean.

**Figure 11.**
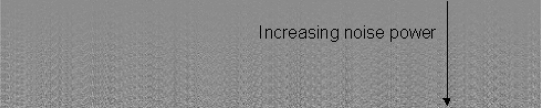
Synthetic image test

A synthetic test image has been generated having the following properties: it is composed by a succession of stripes having equal brightness but different noise power. Each stripe is composed of 10 lines and noise is added with increasing power starting from the top of the image and proceeding downwards (Figure 11).

**Figure 12.**
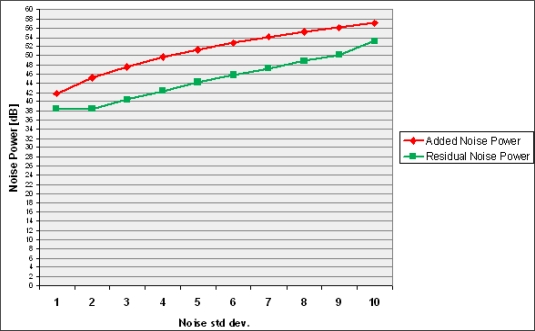
Noise power test. Upper line: noise level before filtering. Lower line: residual noise power after filtering.

The graph in Figure 12 illustrates the filtering effects in terms of noise power; the *x*-axis represents the noise standard deviation; the *y*-axis shows the corresponding noise power decibels before and after filtering. The filter significantly reduces noise and gains up to 6–7dB can be obtained in terms of noise power reduction.

### 4.2. Visual Quality Test

In order to assess the visual quality of the proposed method, we have compared it with the *SUSAN* (Smallest Univalue Segment Assimilating Nucleus) [37] and multistage median filters [36] classical noise reduction algorithm. This choice is motivated by considering the comparable complexity of these solutions. Though more complex recent methods for denoising image data exist [7,8,18,38] achieving very good results, they are not yet suitable for real-time implementation.

**Figure 13.**
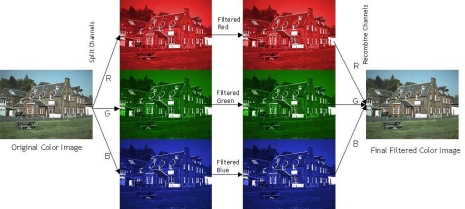
Overall scheme used to compare the Susan algorithm with the proposed method. The noisy color image is filtered by processing its color channels independently. The results are recombined to reconstruct the denoised color image.

The tests were executed using two different approaches. In the first approach, the original noisy Bayer data were interpolated obtaining a noisy color image, which was splitted in its color channels; each color plane was filtered independently using SUSAN. Finally, the filtered color channels were recombined to obtain the denoised color image as sketched in Figure 13.

The second approach consists in slightly modifying the *SUSAN* algorithm so that it can process Bayer data. In both cases, the results of *SUSAN* were compared with the color-interpolated image obtained from a denoised Bayer pattern produced by the proposed method.

**Figure 14.**
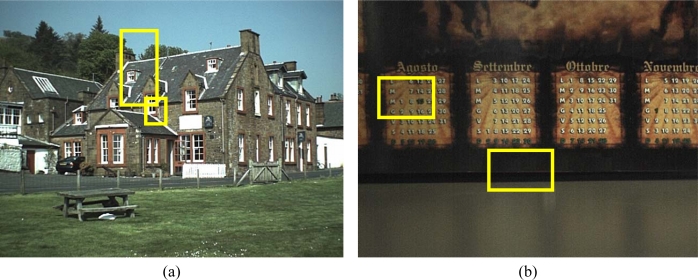
Images acquired by a CFA sensor. (a) SNR value 30.2dB. (b) SNR value 47.2dB. The yellow crops represent the magnified details contained in the following figures.

**Figure 15.**
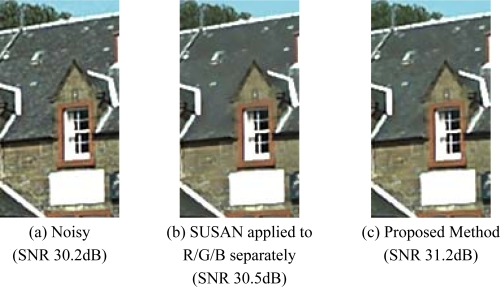
A magnified detail of Figure 14(a), to better evaluate the comparison between the proposed filter and the SUSAN algorithm applied on R/G/B channels separately. Both methods preserve details very well, although the proposed technique is capable to better preserve texture sharpness; the enhancement is visible by looking at the wall and the roof texture. The proposed method uses fewer resources as the whole filtering action takes place on one plane of CFA data.

**Figure 16.**
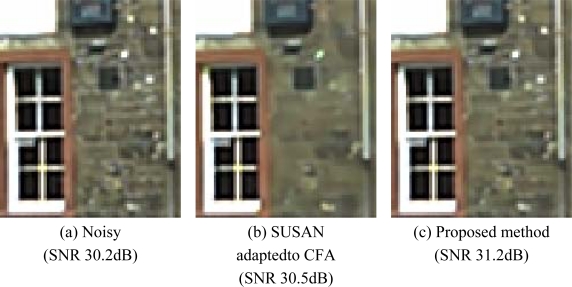
Comparison test at CFA level (magnified details of Figure 14(a)). The original SUSAN implementation was slightly modified so that it can process Bayer data. The efficiency of the proposed method in retaining image sharpness and texture is clearly visible.

**Figure 17.**
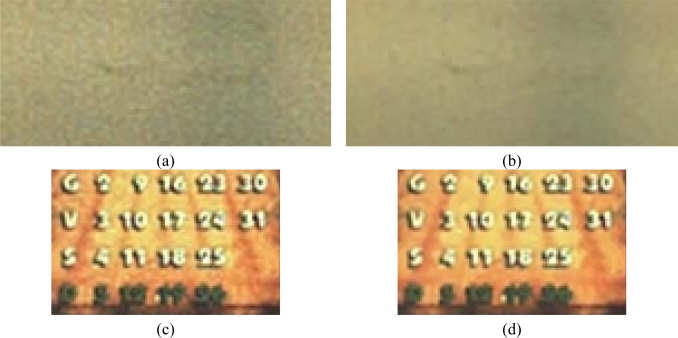
Magnified details of Figure 14(b). (a) 200% zoomed (pixel resize) cropped part of noisy image. (b) Filtered 200% zoomed (pixel resize) counterpart (c) 200% zoomed (pixel resize) cropped part of noisy image. (d) Filtered 200% zoomed (pixel resize) counterpart. The effects of the proposed method over flat (a), (b) and textured (c), (d) areas are shown. The noisy images are obtained by color interpolating unfiltered Bayer data (a), (c). The corresponding color images produced by demosaicing filtered Bayer data (b), (d). SNR values are: 47.2dB for noisy image and 51.8dB for filtered image.

Figure 14 shows two of test noisy reference images acquired by a *CFA* image sensor (2 megapixels) after colour interpolation. Original *SNR* values for the two images are 30.2 dB and 47.2 dB, respectively. After filtering, the corresponding *SNR* values became comparable and higher for both, *SUSAN* and our filtering. In the first comparison test, both algorithms show very good performances; the proposed method, anyway, is capable to preserve some small details that are lost by *SUSAN* independent *R/G/B* filtering. Furthermore, processing is very fast because the method processes only one plane of image information, i.e. the *CFA* data. Figure 15 shows a magnified detail of Figure 14(a) and the filtering results with *SUSAN* and our method. Figure 16 shows how the proposed method significantly retains texture and sharpness after filtering. Figure 17 shows two different details of the noisy image in Figure 14(b) and their filtered counterparts. The homogeneous areas are heavily filtered (a), (b); on the other hand, in textured areas, the detail is well preserved (c), (d).

**Figure 18.**
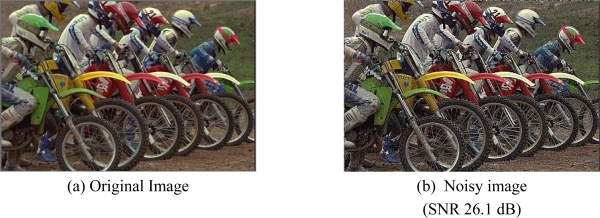

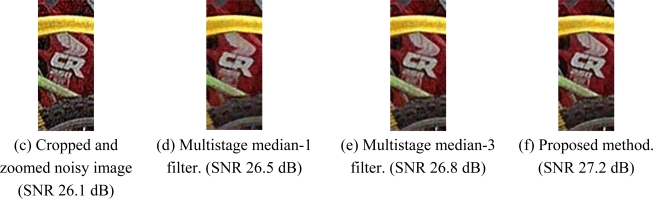
(a) Original Image. (b) Noisy image. (c) Cropped and zoomed noisy image detail. Cropped and zoomed noisy image detail filtered with: Multistage median-1 filter(d), Multistage median-3 filter (e), proposed method(f).

Finally, Figure 18 illustrates the results of the multistage median filters described in [36] compared with the proposed filter. Specifically, the multistage median-1 and multistage median-3 filter outputs were considered. The three methods work on *CFA* data. Figure 18 (e) shows, again, that the proposed filtering technique is able to preserve texture and sharpness very well.

### 4.3. PSNR test

In order to numerically quantify the performance of the filtering process, the standard Kodak 24 (8-bpp) [39] images have been processed with the proposed method comparing them with the outputs of *SUSAN* [37], *Multistage median-1*, *Multistage median-3* algorithms [36] and the following fuzzy approaches from [25]:

- GMED: Gaussian Fuzzy Filter with Median Center
- GMAV: Gaussian Fuzzy Filter with Moving Average Center
- ATMED: Asymmetrical Triangular Fuzzy Filter with Median Center
- ATMAV: Asymmetrical Triangular Fuzzy Filter with Moving Average Center

**Figure 19.**
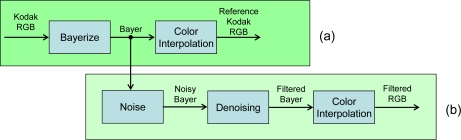
Testing procedure. (a) The original Kodak color image is converted to Bayer pattern format and demosaiced. (b) Noise is added to the Bayer image, filtered and color interpolated again. Hence, color interpolation is the same for the clean reference and the denoised images.

After converting each image of the set to Bayer pattern format, the simulation was performed by adding noise with increasing standard deviation to each CFA plane. In particular the following values have been used: σ = 5, 8, 10. More specifically, the aforementioned values of σ refer to the noise level in the middle of the dynamic range. To simulate a more realistic sensor noise, in fact, we followed the model described in [34,35], that allows obtaining lower noise values for dark areas and higher noise values for bright areas, according to a square root characterization of the noise. In order to exclude the effects of different color interpolations from the computation of the PSNR, the reference images were obtained following the procedure described in Figure 19(a); in this way, both images (i.e. clean and noisy) are generated using the same color interpolation algorithm.

**Figure 20.**
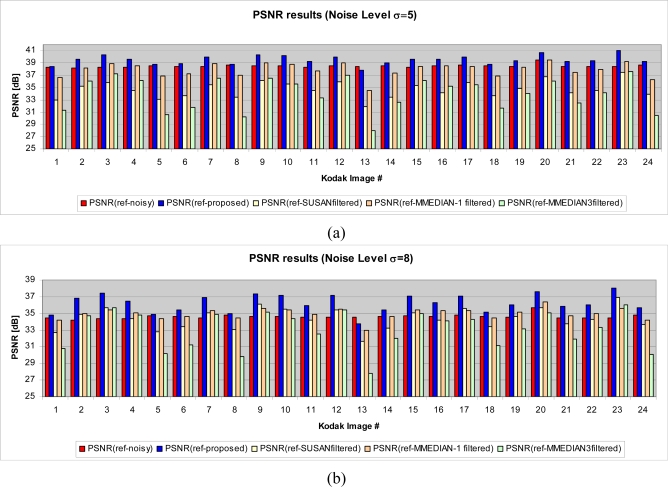

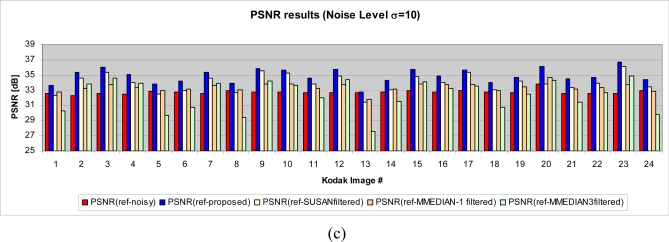
PSNR comparison between proposed solution and other spatial approaches for the Standard Kodak Images test set. (a) Kodak noisy images set with standard deviation 5. (b) Kodak noisy images set with standard deviation 8. (c) Kodak noisy images set with standard deviation 10.

**Figure 21.**
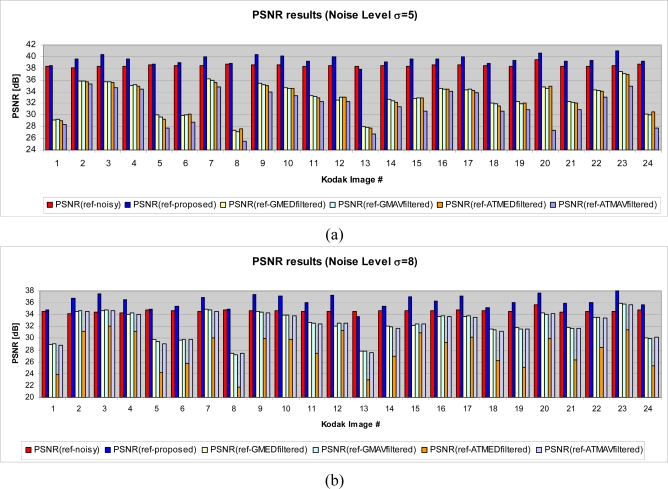

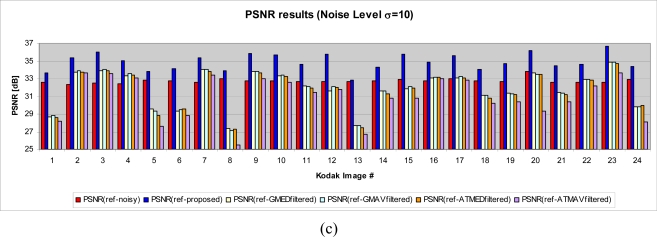
PSNR comparison between proposed solution and other fuzzy approaches for the Standard Kodak Images test set. (a) Kodak noisy images set with standard deviation 5. (b) Kodak noisy images set with standard deviation 8. (c) Kodak noisy images set with standard deviation 10.

Experiments show that the proposed method performs well in terms of PSNR compared to the algorithms used in the test (Figure 20). In order to compare the proposed method with other fuzzy approaches, we considered some methods described in [25]. The results are shown in Figure 21.

## Conclusions and Future Work

A spatial adaptive denoising algorithm has been presented; the method exploits characteristics of the human visual system and sensor noise statistics in order to achieve pleasant results in terms of perceived image quality. The noise level and texture degree are computed to adapt the filter behaviour to the local characteristics of the image. The algorithm is suitable for real time processing of images acquired in CFA format. Future work includes the extension of the processing masks along with the study and integration of other *HVS* characteristics.

## References

[1] Lukac R. (2006). Single-sensor imaging in consumer digital cameras: a survey of recent advances and future directions. J. Real-Time Image Process.

[2] Battiato S., Castorina A., Mancuso M. (2003). High Dynamic Range Imaging for Digital Still Camera: an Overview. SPIE J. Electron. Imaging.

[3] Messina G., Battiato S., Mancuso M., Buemi A. (2002). Improving Image Resolution by Adaptive Back-Projection Correction Techniques. IEEE Trans. Consum. Electron.

[4] Battiato S., Bosco A., Castorina A., Messina G. (2004). Automatic Image Enhancement by Content Dependent Exposure Correction. EURASIP J. Appl. Signal Process.

[5] Battiato S., Castorina A., Guarnera M., Vivirito P. (2003). A Global Enhancement Pipeline for Low-cost Imaging Devices. IEEE Trans. Consum. Electron.

[6] Bayer B.E. (1976). Color Imaging Array. US. Pat. 3,971,965.

[7] Hirakawa K., Parks TW. Joint demosaicing and denoising.

[8] Hirakawa K., Parks T.W. (2006). Joint demosaicing and denoising. IEEE Trans. Image Process.

[9] Lu W., Tan Y.P. (2003). Color Filter Array Demosaicking: New Method and Performance Measures. IEEE Trans. Image Process.

[10] Trussel H., Hartwig R. (2002). Mathematics for Demosaicking. IEEE Trans. Image Process.

[11] Battiato S., Mancuso M. (2001). An Introduction to the Digital Still Camera Technology. ST J. Syst. Res. — Special Issue on Image. Process. Digital Still Camera.

[12] Jayantn N., Johnston J., Safranek R. (1993). Signal Compression Based On Models Of Human Perception. Proceedings of the IEEE.

[13] Nadenau M.J., Winkler S., Alleysson D., Kunt M. (2003). Human Vision Models for Perceptually Optimized Image Processing - a Review. IEEE Trans. Image Process.

[14] Pappas T.N., Safranek R.J., Bovik A.C. (2000). Perceptual Criteria for Image Quality Evaluation. Handbook of Image and Video Processing.

[15] Wang Z., Lu L., Bovik A. Why Is Image Quality Assessment so difficult?.

[16] Wang Z., Bovik A.C., Sheikh H.R., Simoncelli E.P. (2004). Image quality assessment: from error visibility to structural similarity. IEEE Trans. Image Process.

[17] Longere P., Xuemei Z., Delahunt P.B., Brainard D.H. Perceptual Assessment of Demosaicing Algorithm Performance.

[18] Pizurica A., Zlokolica V., Philips W. Combined wavelet domain and temporal denoising.

[19] Portilla J., Strela V., Wainwright M.J., Simoncelli E.P. (2003). Image Denoising Using Scale Mixtures of Gaussians in the Wavelet Domain. IEEE Trans. Image Process.

[20] Scharcanski J., Jung C.R., Clarke R.T. (2002). Adaptive Image Denoising Using Scale and Space Consistency. IEEE Trans. Image Process.

[21] Barcelos C.A.Z., Boaventura M., Silva E.C. (2003). A Well-Balanced Flow Equation for Noise Removal and Edge Detection. IEEE Trans. Image Process.

[22] Amer A., Dubois E. (2005). Fast and reliable structure-oriented video noise estimation. IEEE Trans. Circuits Syst. Video Technol.

[23] Kim Y.-H., Lee J. (2005). Image feature and noise detection based on statistical hypothesis tests and their applications in noise reduction. IEEE Trans. Consum. Electron.

[24] Russo F. (2006). Technique for Image Denoising Based on Adaptive Piecewise Linear Filters and Automatic Parameter Tuning. IEEE Trans. Instrum. Meas.

[25] Kwan H.K., Cai Y. Fuzzy filters for image filtering.

[26] Schulte S., De Witte V., Kerre E.E. (2007). A fuzzy noise reduction method for colour images. IEEE Trans. Image Process.

[27] Bosco A., Findlater K., Battiato S., Castorina A. (2003). Noise Reduction Filter for Full-Frame Imaging Devices. IEEE Trans. Consum. Electron.

[28] Wandell B. (1995). Foundations of Vision.

[29] Gonzales R., Woods R. (1992). Digital Image Processing.

[30] Lian N., Chang L., Tan Y.-P. Improved color filter array demosaicking by accurate luminance estimation.

[31] Chou C-H., Li Y.-C. (1995). A perceptually tuned subband image coder based on the measure of just-noticeable-distortion profile. IEEE Trans. Circuits Syst. Video Technol.

[32] Hontsch I., Karam L.J. (2000). Locally adaptive perceptual image coding. IEEE Trans. Image Process.

[33] Zhang X.H., Lin W.S., Xue P. (2005). Improved estimation for just-noticeable visual distortion. Signal Process.

[34] Foi A., Alenius S., Katkovnik V., Egiazarian K. (2007). Noise measurement for raw-data of digital imaging sensors by automatic segmentation of non-uniform targets. IEEE Sensors J.

[35] Foi A., Trimeche M., Katkovnik V., Egiazarian K. (2008). Practical Poissonian-Gaussian Noise Modeling and Fitting for Single-Image Raw-Data. IEEE Trans. Image Process.

[36] Kalevo O., Rantanen H. Noise Reduction Techniques for Bayer-Matrix Images.

[37] Smith S.M., Brady J.M. (1997). SUSAN - A New Approach to Low Level Image Processing. Int. J. Comput. Vision.

[38] Zhang L., Wu X., Zhang D. (2007). Color Reproduction from Noisy CFA Data of Single Sensor Digital Cameras. IEEE Trans. Image Process.

[39] Standard Kodak test images.

